# Phenotypic Characterization and Antibiotic Resistance Patterns of Extended-Spectrum *β*-Lactamase- and AmpC *β*-Lactamase-Producing Gram-Negative Bacteria in a Referral Hospital, Saudi Arabia

**DOI:** 10.1155/2019/6054694

**Published:** 2019-06-26

**Authors:** Mutasim E. Ibrahim, Mohammed Abbas, Abdullah M. Al-Shahrai, Bahaeldin K. Elamin

**Affiliations:** ^1^Department of Basic Medical Science (Microbiology Unit), College of Medicine, University of Bisha, Bisha, Saudi Arabia; ^2^Department of Pediatrics, College of Medicine, University of Bisha, Bisha, Saudi Arabia; ^3^Department of Family Medicine, College of Medicine, University of Bisha, Bisha, Saudi Arabia; ^4^Department of Medical Microbiology, Faculty of Medical Laboratory Sciences, University of Khartoum, Khartoum, Sudan

## Abstract

**Background:**

Emergence of pathogenic bacteria carrying *β*-lactamase-resistant determinants has become a major health problem in the hospital setting. The study aimed to determine antibiotic-resistant patterns and frequency of extended-spectrum *β*-lactamase- (ESBL-) producing Gram-negative bacteria (GNB) and AmpC *β*-lactamase-producing GNB.

**Methodology:**

A prospective cross-sectional study was conducted during a period from September 2017 to August 2018 at King Abdullah Hospital, Bisha Province, Saudi Arabia. GNB (*n* = 311) were recovered from patients' clinical specimens including sputum, urine, wound pus, blood, tracheal aspirates and high vaginal swabs, umbilical discharge, eye discharge, and cerebrospinal fluids. Isolates were identified by the Phoenix identification system. Antimicrobial susceptibility was tested by the Kirby–Bauer disk procedure. Phenotypic characterization of ESBLs and AmpC *β*-lactamases was performed utilizing the double-disk synergy test and inhibitor-based method, respectively. Associations with outcome measures were determined by simple descriptive statistics and a chi-square test.

**Results:**

Out of 311 GNB isolates, the frequency of ESBL and AmpC *β*-lactamase producers was 84 (27%) and 101 (32.5%), respectively. *Klebsiella pneumoniae* and *Escherichia coli* were common ESBL producers. AmpC *β*-lactamases predominate among *Acinetobacter* spp. and *Pseudomonas aeruginosa*. Coproduction of ESBLs and AmpC *β*-lactamases was found in 36 (11.6%) isolates, with very close relative frequencies among *K. pneumoniae*, *Acinetobacter* spp., and *P. aeruginosa*. *β*-Lactamase producers were predominantly found in the surgical department (56.5%) and ICUs (44.2%). ESBL producers revealed high resistance for cefuroxime (96.4%), cefotaxime (92.9%), and trimethoprim/sulfamethoxazole (90.5%). The resistance rates were significantly higher among ESBL producers than nonproducers for cephalosporins (*p* < 0.001), amoxicillin/clavulanate (*p* < 0.001), piperacillin/tazobactam (*p* = 0.010), nitrofurantoin (*p* = 0.027), aztreonam (*p* < 0.001), ciprofloxacin (*p* = 0.002), and trimethoprim/sulfamethoxazole (*p* < 0.001). Significantly higher (*p* < 0.05) resistance rates were observed among AmpC *β*-lactamase producers than nonproducers for all tested antibiotics.

**Conclusions:**

This finding showed a high prevalence of ESBL- and AmpC *β*-lactamase-producing GNB in our hospital. Quality control practice and routine detection of *β*-lactamase producers before deciding on antibiotic therapy are advocated.

## 1. Introduction

The controlling of infectious diseases caused by pathogenic bacteria has become challenged in the last years due to the extension of bacterial resistance to several antibiotics [1]. Infections caused by bacteria carrying resistant determinants have been associated with increased rates of mortality, hospital stay, therapeutic failure, and health costs [2, 3]. Antibacterial agents of the *β*-lactam group are frequently prescribed medications for the treatment of infections caused by Gram-negative bacteria (GNB) [4]. Members of GNB can hydrolyze many *β*-lactam antibiotics through the production of one or both of extended-spectrum *β*-lactamases (ESBLs) and AmpC *β*-lactamases [3, 4]. The production of ESBLs and AmpC *β*-lactamases mediated by both chromosomal and plasmid genes can transfer horizontally between GNB members [5, 6]. Bacterial strains carrying such enzymes are capable of being resistant to a wide variety of antibiotics, including *β*-lactam drugs [3]. AmpC *β*-lactamases are clinically significant cephalosporinases encoded on chromosomes of Gram-negative rods which mediate resistance to cefoxitin, cephalothin, cefazolin, most of the penicillins, and *β*-lactamase inhibitor [7].

Studies of the antimicrobial susceptibility of GNB revealed an increased resistance due to hyperproduction of ESBL and AmpC enzymes over time [1, 8, 9]. This phenomenon was observed commonly in *E. coli*, *K. pneumoniae*, and other GNB as well [9]. Increasing antibiotic resistance due to the hyperproduction of AmpC enzymes among *Enterobacter* and *Citrobacter* has been reported in Europe [9]. One study in the United States reported a high incidence of ESBL- and AmpC-resistant genes among *E. coli* and *Klebsiella* spp. [10]. In countries of the Gulf Cooperation Council, the high prevalence of ESBL-producing GNB associated with nosocomial infections has been well established [11]. Although considerable studies in Saudi Arabia have been focused on epidemiology and resistant traits of ESBL-producing microorganisms [1, 12, 13], such data are still limited about AmpC *β*-lactamase producers [14]. However, the mechanisms that underpin antibiotic resistance are not thoroughly investigated in certain areas [14, 15]. Routine phenotypic detection of *β*-lactamases carrying resistant strains would be a useful guide for antibiotic therapy and minimizes spreading of these bacteria in hospital settings [4, 5]. This study, therefore, set out to determine resistance patterns and the frequency of ESBL- and AmpC *β*-lactamase-producing GNB from patients at King Abdullah Hospital, Bisha Province, southwest of Saudi Arabia.

## 2. Materials and Methods

### 2.1. Study Design and Setting

A prospective cross-sectional study was conducted between September 2017 and August 2018 at King Abdullah Hospital, Bisha Province, Saudi Arabia. This hospital is a referral hospital with 365 beds distributed into different specialized units [15]. Various clinical specimens were collected from patients of all age groups and submitted to the hospital microbiology laboratory for routine microbiological investigations. The specimens were sputum, urine, wound pus, blood, tracheal aspirates and high vaginal swabs, umbilical discharge, eye discharge, and cerebrospinal fluids. Ethical approval was obtained from the research and ethical committee, College of Medicine, University of Bisha.

### 2.2. Identification of Pathogens

Isolation and identification of GNB were carried out based on cultural characteristics, Gram stains, oxidase test, and conventional biochemical tests following standard assay [16]. Then, full identification of isolate was performed using Phoenix system identification method (Becton, Dickinson, USA). The Phoenix panels were inoculated according to the manufacturer's instructions. Depending on the site of infections and types of specimens, significant growth of each pathogen was identified and processed for antimicrobial susceptibility testing. Every single significant growth of GNB was included in this study. Clinical samples with missed patient personal information and/or yielded more than two isolates were being excluded from the study.

### 2.3. Antimicrobial Susceptibility Testing

Susceptibility testing of the GNB was examined by the Kirby–Bauer disk diffusion assay on Mueller-Hinton agar medium (Oxoid, England) against 18 antibiotic disks following the Clinical and Laboratory Standard Institute guidelines [17]. The following antibiotics were examined: amikacin (30 *μ*g), amoxicillin/clavulanate (20/10 *μ*g), aztreonam (30 *μ*g), cefepime (30 *μ*g), cefotaxime (30 *μ*g), cefoxitin (30 *μ*g), ceftazidime (30 *μ*g), cefuroxime (30 *μ*g), ciprofloxacin (5 *μ*g), colistin (10 *μ*g), gentamicin (10 *μ*g), imipenem (10 *μ*g), meropenem (10 *μ*g), nitrofurantoin (50 μg), piperacillin(100 *μ*g), piperacillin/tazobactam (100/10 *μ*g), tobramycin (10 *μ*g), and trimethoprim/sulfamethoxazole (23.75 *μ*g/1.25 *μ*g) (Oxoid, England). In brief, standardized suspension of each isolate conforming 0.5 McFarland turbidity was inoculated onto two Mueller-Hinton agar plates. Then, nine antibiotic disks were placed onto each plate with recommended distance, followed by overnight incubation at 37°C. The strain of *E. coli* ATCC 25922 was used as control and was tested each time when susceptibility testing was performed.

### 2.4. Detection of ESBL- and AmpC *β*-Lactamase-Producing Bacteria

#### 2.4.1. Double-Disk Synergy Test (DDST)

The DDST was performed to detect ESBL producers as described by Jarlier et al. [18]. The test was performed immediately along with susceptibility testing of each isolate. A susceptibility disk containing amoxicillin/clavulanate (20/10 *μ*g) was placed in the center of the plate. Three disks of cephalosporin agents, namely, ceftazidime (30 *μ*g), cefotaxime (30 *μ*g), and cefepime (30 *μ*g), were located 30 mm apart (center to center) from the amoxicillin/clavulanate disk. All cultured plates were aerobically incubated overnight at 37°C. A visible distortion or extension of the edge of the inhibition zone of cephalosporin towards amoxicillin/clavulanate was interpreted as positive for the production of ESBLs. Strains of *E. coli* ATCC 25922 and *Klebsiella pneumoniae* ATCC 700603 were served as a negative and positive control, respectively.

#### 2.4.2. Inhibitor-Based Method

AmpC *β*-lactamase production was detected by an inhibitor-based method on disk containing boronic acid as previously described [19]. Isolates showed inhibition with zone diameters less than 18 mm for cefoxitin disk (30 *μ*g), which was processed for confirmation of AmpC production. A 0.5 McFarland suspension of tested isolates was inoculated evenly on a Mueller-Hinton agar plate (Oxoid, England). Two disks of cefoxitin (30 *μ*g) with and without boronic acid (400 *μ*g) were placed onto the surface of the plate at a distance of 30 mm. After overnight incubation at 37°C aerobically, a zone of 5 mm or greater around the disk of cefoxitin containing boronic acid compared to the cefoxitin disk was considered for AmpC *β*-lactamase production.

### 2.5. Statistical Analysis

Data management and analysis were performed using the Statistical Package for Social Sciences (SPSS; Version 16.0). Simple descriptive statistics were presented to analyze the outcome data. A chi-square test was used to compare between the resistant patterns of *β*-lactamase- and non-*β*-lactamase-producing isolates. All values less than 0.05 were considered as statistically significant.

## 3. Results

### 3.1. Distribution of Isolates from Clinical Specimens

**Figure 1:**
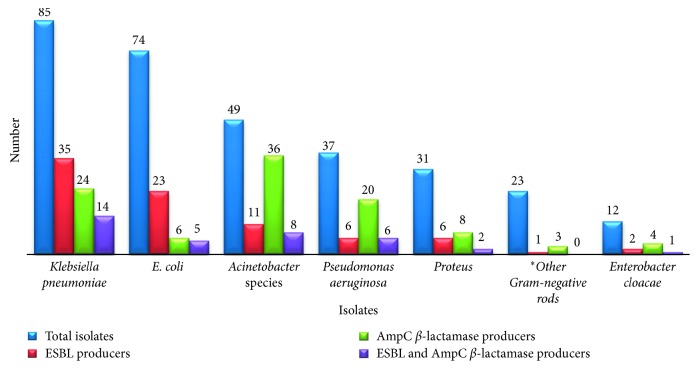
Frequency of ESBLs and AmpC *β*-lactamases, among different Gram-negative pathogens collected from patients at King Abdullah Hospital, Bisha, Saudi Arabia. ^*∗*^*Morganella morganii* (*n* = 5), *Klebsiella oxytoca* (*n* = 4), *Serratia marcescens* (*n* = 4), *Providencia rettgeri* (*n* = 4), *Citrobacter freundii* (*n* = 3), *Salmonella enterica* (*n* = 2), and *Proteus vulgaris* (*n* = 1).

Three hundred eleven GNB were collected from clinical specimens of patients (171 females and 140 males) during the study period. The GNB were obtained from specimens of sputum (*n* = 105), urine (*n* = 99), wound swabs (*n* = 61), blood (*n* = 29), tracheal aspirates and high vaginal swabs (*n* = 5 for each), umbilical swabs (*n* = 3), and eye swabs and cerebrospinal fluids (*n* = 2 for each). *K. pneumoniae* (*n* = 85) was a predominate isolate, followed by *E. coli* (*n* = 74) and *Acinetobacter* spp. (*n* = 49) (Figure 1).

### 3.2. Prevalence of ESBLs and AmpC *β*-Lactamases

**Figure 2:**
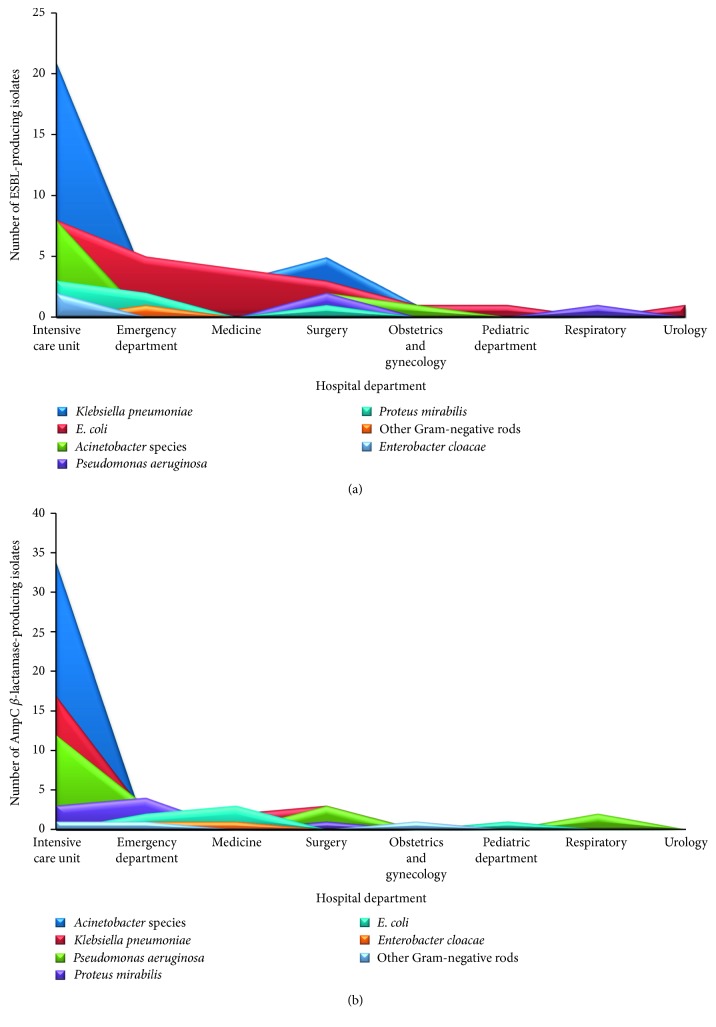
Distribution of different Gram-negative pathogens producing ESBLs (*n* = 84) (a) and AmpC *β*-lactamases (*n* = 101) (b) among hospital departments. High frequency of ESBLs and AmpC *β*-lactamases was shown among *K. pneumoniae* and *Acinetobacter* spp., respectively.

Of the 311 GNB examined for *β*-lactamases, 27% (84) were found to be ESBL producers and 32.5% (101) were AmpC *β*-lactamase producers. ESBL producers were commonly recovered from the surgical department (56.5%), followed by ICUs (29.2%) and medicine department (29.2%) (Table 1 and Figure 2), whereas AmpC *β*-lactamase producers were more frequent in ICUs (44.2%) followed by surgical (34.8%) and medicine (29.2%) departments (Table 2 and Figure 2).

As shown in Figure 1, *K. pneumoniae* was the common ESBL producers followed by *E. coli*, whereas AmpC *β*-lactamases were found more frequently among *Acinetobacter* spp., *P. aeruginosa*, and *Enterobacter cloacae*. Coexistence of ESBLs and AmpC *β*-lactamases was found among 11.6% (36/275) of the isolates. *K. pneumoniae*, *Acinetobacter* spp., and *P. aeruginosa* showed very close relative frequencies with respect to coexistence of ESBLs and AmpC, namely, 16.5%, 16.3%, and 16.2%, respectively.

### 3.3. Resistant Patterns

ESBL producers demonstrated the highest resistance rates to cefuroxime (96.4%), cefotaxime (92.9%), trimethoprim/sulfamethoxazole (90.5%), and cefepime (89.3%). The highest rates of resistance among non-ESBL producers were recorded for trimethoprim/sulfamethoxazole (61.7%) and cefuroxime (60.4%). The resistance rates were significantly higher among ESBL-producing GNB than non-ESBL producers for cephalosporins (*p* < 0.001), amoxicillin/clavulanate (*p* < 0.001), piperacillin/tazobactam (*p*=0.010), nitrofurantoin (*p*=0.027), aztreonam (*p* < 0.001), ciprofloxacin (*p*=0.002), and trimethoprim/sulfamethoxazole (*p* < 0.001) (Table 3).

AmpC *β*-lactamase-producing GNB showed high resistance rates to amoxicillin/clavulanate (92.1%), cefuroxime (91.1%), trimethoprim/sulfamethoxazole (90.1%), and aztreonam (81.2). Non-AmpC-producing GNB showed high susceptibility rates to the most tested antibiotics, except to cefuroxime (60%) and trimethoprim/sulfamethoxazole (59.5%). The resistance rates were significantly (*p* < 0.05) higher among AmpC *β*-lactamase-producing GNB than non-AmpC *β*-lactamase producers for all the tested antibiotics (Table 3).

## 4. Discussion

*β*-Lactamase production among pathogenic bacteria is becoming important resistance mechanisms in hospitals worldwide [3, 4]. There are no current data about the existences of ESBL- and AmpC *β*-lactamase-producing GNB in King Abdullah Hospital, Bisha, Saudi Arabia. In the present study, *β*-lactamase-producing bacteria were commonly found in ICU and surgical departments. This finding is similar to that reported in the eastern region of Saudi Arabia [20] and many parts of the world, such as in Algeria [21] and Nigeria [22]. It has been suggested that the higher use of invasive devices and the selective pressure of newer *β*-lactams for patients at ICU and surgery unit result in the emergence of such pathogens [20]. Noteworthy, GNB collected from the hospital units tend to be *β*-lactamase producers. This might be due to clonal spreading and transmission of *β*-lactamase genes between GNB in the hospital. However, this hypothesis could be to understand by analyzing the clonal similarity of bacterial isolates collected from different hospital wards using specific molecular markers.

In the present study, the prevalence of ESBL-producing GNB was 27%, which is almost similar to that found in the eastern region of the country (30.6%) [12]. However, our result was lower than 72% reported at a tertiary care hospital in Riyadh capital [1]. High prevalence of ESBL-producing bacteria has been observed in many African and Asian countries, such as 47.6% in Algeria [21], 60% in Pakistan [8], and 65% in Nigeria [22]. By contrast, the lowest proportions of ESBL-producing *Enterobacteriaceae* have been reported in Europe, such as below 1% in Sweden [23] and 5% in Netherlands [24]. These reports coupled with the current findings indicated the global dissemination of *β*-lactamase-producing microorganisms, notably in developing countries including Saudi Arabia. This could be attributed to lack of antibiotic policy, poor hygiene conditions in developing countries [25]. Moreover, increasing global trade and international travel were found to be significant risk factors for emerging these resistant bacteria [5]. Indeed, Saudi Arabia has become a significant place for spreading of ESBL microorganisms due to the hosting of mass gathering during Haj and Umrah and population flow from many parts of the world [26]. Therefore, local and national surveillance coupled with the international effort to compact spreading of *β*-lactamase-producing microorganisms is needed.

In the present study, *K. pneumoniae* and *E. coli* were the major ESBL producers although diverse GNB expressed ESBL production. This finding is consistent with previous studies in Saudi Arabia [12, 20, 25]. Likewise, studies in African countries found that *K. pneumoniae* and *E. coli* were the leading ESBL producers in the hospital settings [4, 27].

AmpC *β*-lactamase-producing bacteria may cause nosocomial outbreaks in hospital settings and leading to affect therapeutic choices [28]. The present study showed a higher prevalence of AmpC *β*-lactamase producers (32.5%) compared to a recent study conducted in Saudi Arabia (5.5%) [14]. However, our finding is in agreement with a report from India, where AmpC phenotype was recorded in 36.5% of *Enterobacteriaceae* in a multicenter study [7]. The highest frequency of AmpC *β*-lactamases in this study was found among *Acinetobacter* spp. (73.5%). As well, high incidence of AmpC enzymes in *Acinetobacter* spp. has been reported in China (72%) [29] and India (60%) [30]. These results with our current findings indicated that regular carrying out of infection control procedures could play an important role to reduce spreading of AmpC *β*-lactamase-producing organisms. However, screening of cefoxitin resistance during routine sensitivity tests can aid in early detection of AmpC *β*-lactamase producers and setting of effective antibiotic therapy [7, 28].

In the present study, GNB showed high resistance rates to several classes of antibiotics. However, *β*-lactamase producers revealed significantly higher resistant rates compared to non-*β*-lactamase producers. This finding broadly supported the work of other authors in Saudi Arabia, linking ESBL production with increasing resistant patterns [1, 20, 31, 32]. In the present study, ESBL-producing GNB displayed an increasing rate of resistance for carbapenem group, such as meropenem (28.6%) and imipenem (31%). These elevated rates were also reported in Riyadh capital by Marie et al. (2013) [1]. Furthermore, AmpC *β*-lactamase-producing GNB demonstrated high rate of resistance to meropenem (49.5%) and imipenem (57.2%). Such figures are of great concern since carbapenems are considered the drugs of choice for therapy of serious ESBL- and AmpC *β*-lactamase-associated infections in the country [20, 33]. The possible explanation for increasing resistance rates might attribute to antibiotic misuse in general and frequent prescription of carbapenems in our hospital. Therefore, phenotypic detection of *β*-lactamase-producing GNB should be carried routinely before antibiotic therapy. However, several phenotypic tests are available, cheap, and easier to conduct routinely in concurrent with routine susceptibility testing of GNB in the hospital laboratory.

In the present study, coproduction of ESBLs and AmpC *β*-lactamases was found among 11.6% of the isolates. This proportion is relatively lower than 14.3% reported among *P. aeruginosa* in Pakistan [34]. Likewise, several studies identified the production of ESBLs and AmpC *β*-lactamases together by GNB [3, 35].

Bacterial strains producing both ESBLs and AmpC *β*-lactamases are often more resistant to *β*-lactam, *β*-lactamase inhibitor combinations, carbapenems, and several antibiotic groups [26]. In this study, GNB carrying ESBLs and AmpC *β*-lactamases revealed high resistant rates to most of the antibiotics. A strong association between plasmid AmpC *β*-lactamases with ESBL, plasmid-mediated quinolone resistance, and aminoglycoside-modifying enzymes has been well documented in the literature [14, 20]. However, the genotypic study of different families of *β*-lactamase-encoded resistant genes might be essential to develop a full picture of *β*-lactamases resistance mechanisms in GNB.

## 5. Conclusion

In conclusion, the present study reported high prevalence of ESBL- and AmpC *β*-lactamase-producing GNB, mainly among *K. pneumoniae*, *E. coli*, and *Acinetobacter* spp. in King Abdullah Hospital. Coexistence of ESBLs and AmpC *β*-lactamases was found among 11.6% of the isolates, making the infection caused by those bacteria more challenging to treat. Escalating levels of antibiotic resistance among ESBL and AmpC *β*-lactamase producers were observed, leaving limited therapeutic options. The presences of ESBLs and AmpC *β*-lactamases were fundamental mechanisms of increasing resistance rates among Gram-negative pathogens in our hospital, although other resistant determinants have not investigated yet. These findings impose that regular carrying out of infection control procedures could play an important role to reduce spreading of ESBL- and AmpC *β*-lactamase-producing isolates. Routine detection of ESBL- and AmpC *β*-lactamase-producing isolates before deciding on antibiotic therapy is advocated.

## Figures and Tables

**Table 1:** Distribution of ESBL-producing Gram-negative bacteria among hospital departments at King Abdullah Hospital, Bisha, Saudi Arabia.

Isolates (*n*)	Total number (%) of ESBL producers	Gram-negative isolates per hospital department							
		ICUs (*n* =154), ESBL+	ICUs (*n* = 154), ESBL+	Medicine (*n* = 24), ESBL+	Surgery (*n* = 23), ESBL+	OBS and gyne (*n* = 22), ESBL+	Pediatric (*n* = 10), ESBL+	Respiratory (*n* = 8), ESBL+	Urology (*n* = 7), ESBL+
*K. pneumoniae* (*n* = 85)	35 (41.2)	21	3	3	5	1	1	0	1
*E. coli* (*n* = 74)	23 (31.1)	8	5	4	3	1	1	0	1
*Acinetobacter* spp. (*n* = 49)	11 (22.4)	8	0	0	2	1	0	0	0
*P. aeruginosa* (*n* = 37)	6 (16.2)	3	0	0	2	0	0	1	0
*Proteus mirabilis* (*n* = 31)	6 (19.4)	3	2	0	1	0	0	0	0
Other Gram-negative rods^*∗*^ (*n* = 12)	1 (4.3)	0	1	0	0	0	0	0	0
*Enterobacter cloacae* (*n* = 12)	2 (16.7)	2	0	0	0	0	0	0	0
Total (*n* = 311)	84 (27)	45 (29.2)	11 (17.5)	7 (29.2)	13 (56.5)	3 (13.6)	2 (20)	1 (12.5)	2 (28.6)

ESBL = extended-spectrum *β*-lactamase; OBS and gyne = obstetrics and gynecology; ICUs = intensive care units. ^*∗*^*Morganella morganii* (*n* = 5), *Klebsiella oxytoca* (*n* = 4), *Serratia marcescens* (*n* = 4), *Providencia rettgeri* (*n* = 4), *Citrobacter freundii* (*n* = 3), *Salmonella enterica* (*n* = 2), and *Proteus vulgaris* (*n* = 1).

**Table 2:** Distribution of AmpC *β*-lactamase-producing Gram-negative bacteria among hospital departments at King Abdullah Hospital, Bisha, Saudi Arabia.

Isolates (*n*)	Total number (%) of AmpC *β*-lactamase producers	Gram-negative isolates per hospital department							
		ICUs (*n* = 154), AmpC+	Emergency (*n* = 63), AmpC+	Medicine (*n* = 24), AmpC+	Surgery (*n* = 23), AmpC+	OBS and gyne (*n* = 22), AmpC+	Pediatric (*n* = 10), AmpC+	Respiratory (*n* = 8), AmpC+	Urology (*n* = 7), AmpC+
*K. pneumoniae* (*n* = 85)	24 (28.2)	17	2	2	3	0	0	0	0
*E. coli* (*n* = 74)	6 (8.1)	0	2	3	0	0	1	0	0
*Acinetobacter* spp. (*n* = 49)	36 (73.5)	34	0	1	1	0	0	0	0
*P. aeruginosa* (*n* = 37)	20 (54.1)	12	3	0	3	0	0	2	0
*Proteus mirabilis* (*n* = 31)	8 (25.8)	3	4	0	1	0	0	0	0
Other Gram-negative rods (*n* = 12)	3 (13.0)	1	1	0	0	1	0	0	0
*Enterobacter cloacae* (*n* = 12)	4 (33.3)	1	1	1	0.0	1	0	0	0
Total (*n* = 311)	101 (32.5)	68 (44.2)	13 (20.6)	7 (29.2)	8 (34.8)	2 (9.1)	1 (10)	2 (25)	0 (0.0)

OBS and gyne = obstetrics and gynecology; ICUs = intensive care units.

**Table 3:** Antibiotic resistance patterns of Gram-negative bacteria producing ESBLs and AmpC *β*-lactamases compared to nonproducer isolates.

Antibiotic	Total number of Gram-negative rods (*n* = 311)		*p* value	Total number of Gram-negative rods (*n* = 311)		*p* value
	Non-ESBL (*n* = 227)	ESBL (*n* = 84)		AmpC (*n* = 101)	Non-AmpC (*n* = 210)	
Amikacin	31 (36.9)	61(26.9)	0.085	57 (56.4)	35 (16.7)	<0.001
Tobramycin	50 (42)	92 (40.5)	0.134	67 (66.3)	67 (31.9)	<0.001
Gentamicin	40 (47.6)	81 (35.7)	0.055	66 (65.3)	55 (26.2)	<0.001
Amoxicillin/clavulanate	58 (69)	91 (40.1)	<0.001	93 (92.1)	56 (26.7)	<0.001
Cefuroxime	81 (96.4)	137 (60.4)	<0.001	92 (91.1)	126 (60)	<0.001
Ceftazidime	72 (85.7)	76 (33.5)	<0.001	65 (64.4)	83 (39.5)	<0.001
Cefotaxime	78 (92.9)	85 (37.4)	<0.001	76 (75.2)	87 (41.4)	<0.001
Cefepime	75 (89.3)	87 (38.3)	<0.001	75 (74.3)	87 (41.4)	<0.001
Meropenem	24 (28.6)	59 (26.0)	0.755	50 (49.5)	33 (15.7)	<0.001
Piperacillin	53 (63.1)	77 (33.9)	<0.001	72 (71.3)	58 (27.6)	<0.001
Piperacillin/tazobactam	42 (50)	77 (33.9)	0.010	65 (64.4)	54 (25.7)	<0.001
Imipenem	26 (31)	62 (27.3)	0.527	58 (57.4)	30 (14.3)	<0.001
Aztreonam	69 (82.1)	98 (43.2)	<0.001	82 (81.2)	85 (40.5)	<0.001
Ciprofloxacin	51 (60.7)	92 (40.5)	0.002	74 (73.3)	69 (32.9)	<0.001
Ofloxacin	50 (59.5)	91 (40.1)	0.002	70 (69.3)	71 (33.8)	<0.001
Nitrofurantoin	50 (59.5)	103 (45.4)	0.027	78 (77.2)	75 (35.7)	<0.001
Trimethoprim/sulfamethoxazole	76 (90.5)	140 (61.7)	<0.001	91 (90.1)	125 (59.5)	<0.001
Colistin	4 (4.8)	5 (2.2)	0232	7 (6.9)	2(1.0)	0.003

## Data Availability

All the data supporting our findings were incorporated within the article. Raw data can be presented by the principal investigator upon request.
